# The miR-199a-5p/PD-L1 axis regulates cell proliferation, migration and invasion in follicular thyroid carcinoma

**DOI:** 10.1186/s12885-022-09838-0

**Published:** 2022-07-11

**Authors:** Jianguang Lin, Yanru Qiu, Xueqin Zheng, Yijun Dai, Tianwen Xu

**Affiliations:** Department of Oncology, The Second Affiliated Hospital of Fujian Medical University, Quanzhou, 362000 Fujian China

**Keywords:** Follicular thyroid carcinoma, miR-199a-5p, PD-L1, Claudin-1, Immune infiltration

## Abstract

**Background:**

Follicular thyroid carcinoma (FTC) is the second most common cancer of the thyroid and easily develops into distant metastasis. PD-L1 is known to be associated with the carcinogenesis and progression of thyroid carcinoma. Our study aimed to investigate the biological functions of PD-L1 and to identify miRNAs that were responsible for modulating the activity of PD-L1.

**Methods:**

A total of 72 patients with FTC at The Second Affiliated Hospital of Fujian Medical University were enrolled in this retrospective study. Immunohistochemical (IHC) assay was used to measure PD-L1 expression in FTC. The association between PD-L1 expression and clinicopathologic characteristics was evaluated. Bioinformatics analysis, RT–qPCR and western blotting were used to examine the relationships between miR-199a-5p, PD-L1 and Claudin-1. Cell proliferation, migration and invasion were evaluated by using CCK8 and Transwell migration and invasion assays. Target prediction and luciferase reporter assays were performed to verify the binding between miR-199a-5p and PD-L1. Rescue assay was performed to confirm whether PD-L1 downregulation abolished the inhibitory effect of miR-199a-5p.

**Results:**

Among 72 pairs of tumor and normal specimens, the proportion of PD-L1 positive samples was higher in FTC tissues than in normal tissues. The results of ESTIMATE and CIBERSORT illustrated that there was a positive correlation between PD-L1 expression and immune infiltration, especially regulatory T cells and M1 macrophages. Prediction of immunotherapy revealed that patients with high PD-L1 expression might benefit from immune checkpoint inhibitors. Transwell migration and invasion assays showed that PD-L1 downregulation in FTC cells could significantly inhibit cell migration and invasion. The bioinformatics analysis and luciferase activity results indicated that PD-L1 was a potential target of miR-199a-5p. Knockdown of PD-L1 reversed the miR-199a-5p inhibitor mediated promotion effect. In addition, we found that PD-L1 expression was positively correlated with Claudin-1 expression and that miR-199a-5p affected the progression of FTC cells through the negative regulation of PD-L1 and Claudin-1.

**Conclusions:**

Our study revealed that PD-L1 expression was elevated in FTC and was closely associated with tumor aggressiveness and progression. MiR-199a-5p has a functional role in the progression and metastasis of FTC by regulating PD-L1 and Claudin-1 expression.

**Supplementary Information:**

The online version contains supplementary material available at 10.1186/s12885-022-09838-0.

## Background

Thyroid carcinoma (TC) is the most prevalent endocrine tumor, and also has one of the most quickly increasing incidences of malignancy tumor worldwide [1]. In general, thyroid carcinoma can be divided into differentiated thyroid carcinoma (DTC), undifferentiated thyroid cancer (ATC) and medullary thyroid carcinoma (MTC) according to histopathological classification [2]. Most thyroid cancers are differentiated cancers that can be further divided into papillary thyroid carcinoma (PTC) and follicular thyroid carcinoma (FTC) [3]. Because of its indolent nature, papillary cancer has excellent long-term survival after complete initial therapy, with a 10-year overall survival greater than 95%. Compared with PTC, FTC is a moderately differentiated cancer with a higher degree of malignancy, which easily develops into distant metastasis. FTC is the next most common thyroid carcinoma compared with PTC, which is easy to develop into distant metastasis. Surgery is the standard therapeutic approach for patients with thyroid carcinoma [4]. FTC is the next most common thyroid carcinoma compared with PTC, which easily develops into distant metastasis. Surgery is the standard therapeutic approach for patients with thyroid carcinoma [4]. However, 10–20% of patients with FTC experience recurrence and metastatic disease after standardized treatment. Current therapeutic strategies for these patients are limited and have poor prognoses. The survival rate for thyroid carcinoma patients with lung or bone metastases was 53% at 5 years [5]. Immunotherapy has emerged as a promising approach for various cancer types, and an increasing number of studies have shown that patients with advanced thyroid cancer can benefit from immunotherapy [6]. However, there are few reports about the regulatory effect of PD-L1 and its potential roles in FTC. In particular, the roles of miRNAs in the regulation of PD-L1 have not been defined. We hope that the content studied in this article can provide useful information and reference value for the treatment of FTC.

PD-L1 is a member of the B7 superfamily, which can be expressed in various types of tumors including lung cancer [7], melanoma [8] and thyroid cancer [9]; it plays a crucial role in the maintenance of immunological tolerance and is associated with poor prognosis and anti-tumor treatment resistance [10]. The expression of PD-L1 is an established prerequisite for immune checkpoint inhibitors in several tumor types [11]. Therefore, researchers considering the potential use of checkpoint inhibitors and PD-L1 expression have mainly focused on these aggressive subsets of thyroid cancers [12]. However, the molecular mechanism underlying PD-L1 expression in FTC remains largely unclear. Therefore, it is of great significance to investigate the expression of PD-L1 in FTC and explore its biological mechanism.

MiRNAs can affect the occurrence and development of tumors as carcinogenic factors or tumor suppressor genes and also affect the immunogenicity and antitumor immune response of tumors [13, 14]. One of the mechanisms of cancer immune evasion includes the overexpression of PD-L1 [15]. Several studies have reported that miRNAs can directly target the 3’-UTR structure of PD-L1 to inhibit the expression of PD-L1 [16]. These miRNAs can affect the function of immune signalling by regulating the expression of PD-L1 and PD-1 and attracting immune cells to the tumor microenvironment [17]. Hence, it is crucial to understand the role of miRNAs and explore their biological function. Claudin-1 belongs to the transmembrane tight junction protein family of epithelial cells and plays an important role in the occurrence and development of tumors. Several studies have shown that upregulation of Claudin-1 is associated with increased cancer cell invasiveness and leads to worse prognosis in follicular thyroid cancer. In addition, Xu et al. showed that PD-L1 expression is closely related to the expression of tight junction proteins in acute lung injury [18]. However, whether miRNAs can regulate the expression of Claudin-1 via PD-L1 in FTC remains unknown.

PD-L1 is a classical immune checkpoint molecule expressed on the surfaces of tumor cells and immune cells and plays a crucial role in the immune system [19]. However, PD-L1 can also influence tumor progression by regulating immune-independent and intrinsic cellular functions [20]. Several studies have shown that PD-L1 can promote cancer progression and metastasis through different signalling pathways [21]. In this study, we sought to assess the relationship between PD-L1 and miRNAs in FTC and investigate their potential functional role in mediating tumor aggressiveness and progression in FTC. These results could provide a better understanding of the molecular mechanisms of cancer progression and metastasis in FTC.

## Methods

### Immunohistochemical (IHC) staining

Seventy-two paired surgically resected FTC tissue and adjacent normal tissue samples were collected from The Second Affiliated Hospital of Fujian Medical University between January 2015 and October 2021. This study was approved by the Ethics Committee of The Second Affiliated Hospital of Fujian Medical University. Immunohistochemical (IHC) staining for PD-L1 was performed with the monoclonal mouse anti-human PD-L1 antibody (Clone: 2B11D11, Proteintech, China) according to recommended protocols. Evaluation of the percentage of tumor cells with partial or complete membranous staining was performed by two pathologists. The tumor proportion score (TPS) was defined as the percentage of tumor cells with complete or partial membranous staining at any intensity. A TPS ≥ 1% was considered positive.

### Cell lines and cell culture

A follicular thyroid carcinoma cell line (FTC-133), a normal thyroid epithelial cell line (Nthy-ori 3–1) and 293 T cells were acquired from Procell Life Science & Technology (Wuhan, China). These cell lines were cultured in DMEM or RPMI 1640 (Gibco, USA) with 10% heat-inactivated foetal bovine serum (FBS) (Gibco, USA), 100 U/mL penicillin, and 100 U/mL streptomycin. All cells were maintained at 37 °C in a humidified 5% CO_2_ atmosphere cell incubator.

### Bioinformatics analysis

The transcriptome and miRNA expression profiles and corresponding clinical information of FTC were downloaded from the Genomic Data Commons Data Portal of TCGA (https://portal.gdc.cancer.gov/, accessed February 1, 2022). A total of 106 specimens were analysed, which included 99 tumors and 6 matched normal tissue specimens. “LIMMA” is an R software package that was used for differential expression analysis of microarray and RNA-seq data. The differentially expressed miRNAs were screened using the “LIMMA” package, with parameters of log fold change (FC) > 1 or < -1 and *P* value < 0.05. Tumor-infiltrating immune cells were evaluated using the CIBERSORT algorithm [22]. CIBERSORT was used to quantify the infiltration of 22 immune cells, including T cells (CD8^+^ T cells, CD4^+^ T cells, resting memory CD4^+^ T cells, naïve CD4^+^ T cells, γδ T cells, regulatory T cells, and follicular helper T cells), B cells (naïve and memory B cells and plasma cells), NK cells (activated and resting NK cells), and myeloid subsets (M0 macrophages, M1 macrophages, M2 macrophages, activated and resting mast cells, activated and resting dendritic cells, neutrophils, monocytes and eosinophils). Only results with *p* value < 0.05 were filtered and selected for the next analysis. The ESTIMATE algorithm was applied to calculate the immune cell infiltration level, including the stromal score, immune score, ESTIMATE score and tumor purity of FTC samples [23]. The Cancer Immunome Atlas (TCIA) is a dataset that provides comprehensive immunogenomic analyses based on TCGA data. We used this database to evaluate the immunophenoscore (IPS) of tumor samples, which can predict the response to CTLA-4 and PD-1 blockers [24].

### Functional enrichment analysis

Kyoto Encyclopedia of Genes and Genomes (KEGG) enrichment analyses were conducted to analyse the enriched pathways between the PD-L1 high and low expression groups by the “ggplot2” and “GSVA” packages [25]. The “ggplot2” and “GSVA” R packages are download from Bioconductor. Gene set variation analysis (GSVA) is a nonparametric, unsupervised method for estimating variation in gene set enrichment through the samples of an expression dataset. Gene set enrichment analysis (GSEA) was conducted using the hallmark c5.go. v 7.5.1 gene sets, based on which GSEA was performed using GSEA software (v4.2.3).

### SiRNA transfection

To knock down the expression of PD-L1, siRNA fragments from the coding regions of three PD-L1 genes (siPD-L1-1, siPD-L1-2, siPD-L1-3) and empty vector siRNA fragments (NC) (Sangon, Shanghai, China) were transfected into FTC-133 cells. Then, 18–24 h before transfection, FTC-133 cells were seeded on a 10 cm culture dish and transfected by lipoRNAi (Beyotime, Shanghai, China) when the cell density reached 70% ~ 80% in complete medium. The culture was continued 24 h after transfection with a new complete medium. Three interfering siRNAs were transfected into FTC-133 cells as the experimental group (siPD-L1 group), and empty vector siRNAs were transfected as the control group (si-NC group). Only LipoRNAi transfection reagent was added to the control group. The target sequences of siPD-L1 are listed in Table S1.

### MiRNA transfection

miRNAs, including miR-199a-5p mimics, miR-199a-5p inhibitor and the nontargeting control (mimics -NC and inhibitor-NC), were obtained from Sangon (Shanghai, China). For cell transfection, miR-199a-5p mimics, miR-199a-5p inhibitor transfected and their negative control (mimics -NC and inhibitor-NC) were transfected into FTC cells with Lipofectamine 3000 reagent (Invitrogen, USA) according to the manufacturer’s instructions. RT–qPCR was used to measure the efficiency of transfection. All nucleotide sequences are listed in Table S2.

### Quantitative reverse transcription PCR (RT–qPCR)

Total RNA in FTC cells was extracted using an RNA Extraction Kit (Beyotime, Shanghai, China) according to the manufacturer’s instructions. Then, reverse transcription of RNA was conducted by using a cDNA Synthesis Kit (TaKaRa, Tokyo, Japan), and RT–qPCR was performed for the obtained cDNA with SYBR Premix Ex Taq (TaKaRa, Tokyo, Japan). GAPDH was used as an internal control for the detection of PD-L1 and Claudin-1, while U6 was used as an internal control for miR-199a-5p. The relative expression was calculated by the 2-ΔΔCT method, and the U6 and GAPDH mRNA expression levels were used as reference genes. The primers were synthesized by Sangon Biotech (Shanghai, China) and are listed in Table S2.

### Western blotting analysis

Protein samples were obtained from cells using RIPA lysis buffer containing a protease and phosphatase inhibitor cocktail. Twenty micrograms of protein was separated using 10% SDS–PAGE and then transferred onto polyvinylidene difluoride (PVDF) membranes. After blocking with blocking buffer at room temperature for 15 min, PVDF membranes were incubated with primary antibodies, including anti-PD-L1 (1:2 000, Proteintech, China), anti-Claudin-1 (1:2 000, Proteintech, China) and anti-GAPDH (1:4 000, Proteintech, China), at 4 °C overnight. Then, they were incubated with the corresponding secondary antibody for 1–2 h at room temperature. The protein bands were visualized using the BeyoECL Moon detection system. For quantitative analysis of protein expression, ImageJ software was applied to measure the optical densities of the blot bands.

### Cell counting Kit-8 (CCK-8 assay)

CCK-8 (Everbright Inc., USA) assay was used to assess the proliferation ability of FTC cells. Twenty-four hours after cell transfection, FTC-133 cells were seeded into 96-well plates at an initial concentration of 1 × 10^4^ cells/mL per well. CCK-8 solution was added to the corresponding wells, and the cells were incubated at 37 °C. Then, the cells were incubated for 0, 24, 48, 72 and 96 h. Cell proliferation was detected by measuring absorbance at a wavelength of 450 nm.

### Migration and invasion assays

Transwell assay was used to examine the migration and invasion ability of the FTC cells. Twenty-four hours after transfection, adherent FTC cells were detached with trypsin and adjusted to 1 × 10^5^/mL with RPMI 1640 medium without foetal bovine serum. In the cell migration assay, 2 × 10^4^ cells with 200 μL serum-free medium were added to the upper chamber without Matrigel, and 600 μL cell culture medium with 10% foetal bovine serum was added to the bottom chamber. For the invasion assay, the upper surface of the upper membrane was first coated with 50 µl Matrigel (BD Bioscience, USA). After incubation for 36 h, the cells were fixed with 4% paraformaldehyde for 30 min and stained with crystal violet for 20 min. Finally, the number of cells in the compartment was counted to determine the average value from five visual fields that were randomly selected under the microscope.

### Dual-luciferase reporter assay

The 3’-UTR fragment of PD-L1 was amplified and cloned into the check2 luciferase vector to construct the wild-type plasmid (WT), which was named PD-L1-3’UTR-WT. The mutant 3’-UTR fragment was obtained by point mutation and inserted into the check2 vector to construct the mutant plasmid (MU), which was named PD-L1-3’UTR-MU. HEK-293 T cells are a human renal epithelial cell line derived from human embryonic kidney cells which were commonly used in dual-luciferase reporter assay for transfection of miRNA. 293 T cells were inoculated in 12-well plates at a density of 2 × 10^5^ cells per well. When the cells reached 50%-70%, 10 µl DMEM was mixed with 5 pmol mimics control (mimics NC) or miR-199a-5p mimics and 0.16 µg PD-L1-3’UTR-WT or PD-L1-3’UTR-MU plasmid and transfected into 293 T cells with Lipofectamine 2000. First, cells were divided into miR-199a-5p mimics transfected with PD-L1-3 ‘UTR-WT and mimics control (mimics NC) transfected with PD-L1-3 ‘UTR-WT. Next, cells were divided into miR-199a-5p mimics transfected with PD-L1-3’UTR-MU and mimics control (mimics NC) transfected with PD-L1-3’UTR-MU. Forty-eight hours after transfection, Renilla and firefly luciferase activities were examined using the dual-luciferase reporter assay system (Promega Corporation, USA).

### Statistical analysis

IBM SPSS Statistics version 23.0 software, GraphPad Prism version 8.0 software and R software were used as statistical tools. The comparisons of categorical data were analysed using the chi-square test. The data of clinical patients are presented as the means ± standard deviations (SD). Correlation analysis was conducted using Spearman correlation. The Wilcoxon test was performed to compare continuous variables between groups in bioinformatic analysis. Comparisons among multiple groups were conducted by the Kruskal–Wallis test. For in vitro experiments, because a normal distribution is not expected, the significant results were calculated by a nonparametric 2-tailed Student’s t test. A *p* value < 0.05 was considered statistically significant.

## Results

### PD-L1 expression is upregulated in FTC tissues and cell lines

**Fig. 1:**
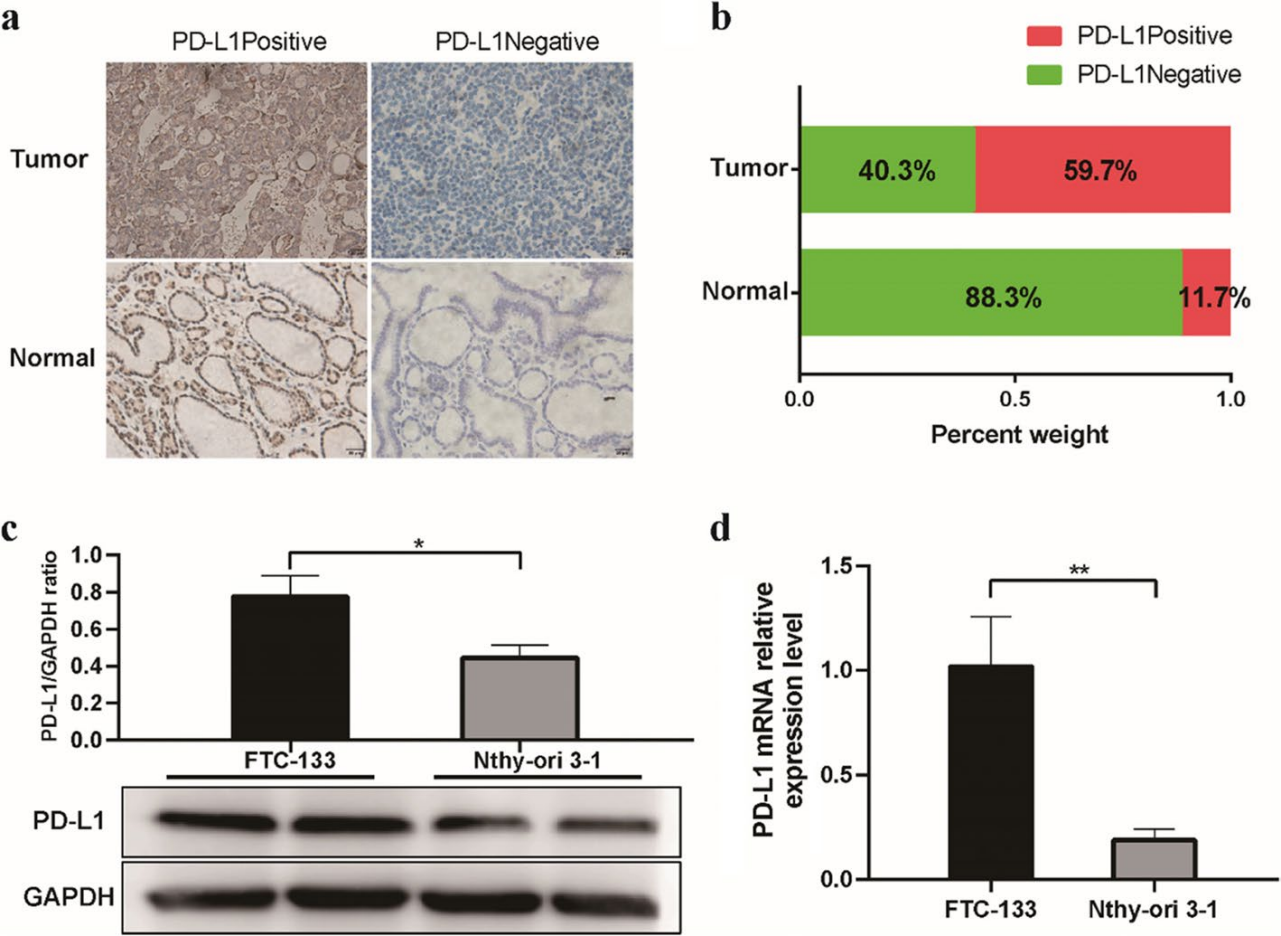
PD-L1 expression is upregulated in FTC tissues and cell lines.** a** The expression of PD-L1 in tumor and adjacent normal tissues was examined by IHC staining. **b** Comparison of PD-L1 expression between tumor and adjacent normal tissues. **c** The levels of PD-L1 protein in FTC-133 and Nthy-ori 3–1 cells were detected by Western blot. **d** The expression levels of PD-L1 mRNA in FTC-133 and Nthy-ori 3–1 cells were examined by RT–qPCR. * *p* < 0.05, ** *p* < 0.01, *** *p* < 0.001

To explore the association between PD-L1 expression and FTC, IHC staining was performed on FTC tissues and corresponding adjacent normal tissues of 72 FTC patients. Among 72 tumor specimens, 43 cases (59.7%) were PD-L1 positive, and 29 cases (40.3%) were PD-L1 negative. We found that the proportion of PD-L1-positive samples was higher in FTC tissues than in normal tissues (Fig. 1a-b). There were positive correlations between PD-L1 expression and tumor size, multifocality, extrathyroid infiltration, vascular invasion and postoperative recurrence (*p* < 0.05, Table 1). No significant differences in other clinicopathological features, such as age, sex, lymph nodes or metastasis were found between the PD-L1-positive and PD-L1-negative groups (*p* > 0.05). Next, we analysed the expression of PD-L1 in the follicular thyroid cancer cell line FTC-133 and the normal thyroid epithelial cell line Nthy-ori 3–1 using western blotting and RT‐qPCR. Consistently, the expression level of PD-L1 in FTC-133 cells was significantly higher than that in Nthy-ori 3–1 cells (Fig. 1c-d).

**Table 1:** Relationship between PD-L1 expression and their clinic pathological characteristics of FTC patients

Clinicopathological feature	NO	PD-L1		x^2^	*P*
		positive	negative		
**Age (a)**					
< 55	55	32	23	0.230	0.779
≥ 55	17	11	6		
**Gender**					
Male	49	31	18	0.801	0.442
Female	23	12	11		
**Size(cm)**					
≤ 4	35	16	19	5.556	0.030^*^
> 4	37	27	10		
**Multifocality**					
Positive	26	20	6	5.005	0.044^*^
Negative	46	23	23		
**Extrathyroid infiltration**					
Positive	16	15	1	9.902	0.003^*^
Negative	56	28	28		
**LN metastasis**					
Positive	9	7	2	1.394	0.297
Negative	63	36	27		
**Vascular invasion**					
Positive	45	31	13	5.418	0.023^*^
Negative	28	12	16		
**TNM stage**					
I-II	60	34	26	0.338	1.397
III-IV	12	9	3		
**Recurrence**					
Yes	15	14	1	8.898	0.003^*^
No	57	29	28		

* *p* < 0.05

### Association of PD-L1 expression and tumor immune infiltration in FTC

**Fig. 2:**
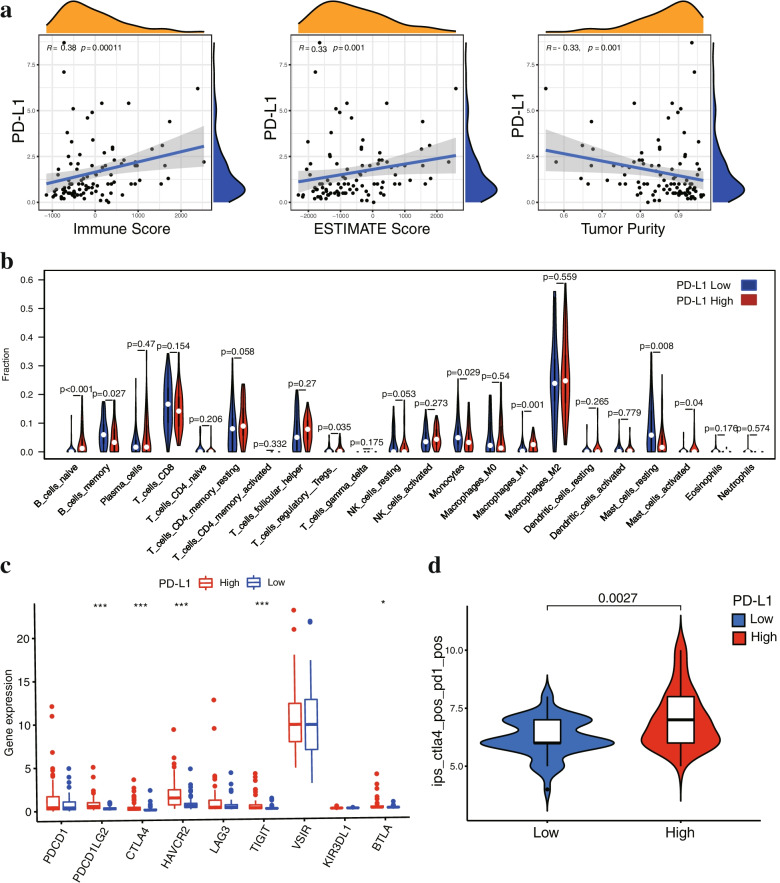
Association of PD-L1 expression and tumor immune infiltration in FTC. **a** Correlation between PD-L1 expression and TME score (immune score and ESTIMATE score) in FTC. **b** Comparisons of immune cells between the PD-L1 high and low expression groups in TCGA. **c** The expression levels of inhibitory checkpoint molecules, including PD-1, PD-L2, CTLA-4, TIM-3, LAG-3, VISTA, TIGIT, KIR and BTLA, between the two groups of FTC patients. **d** The IPS for immune checkpoint inhibitors in the PD-L1 high expression group was significantly higher, suggesting that patients with higher PD-L1 expression would have a better response to immune checkpoint inhibitors. * *p* < 0.05, ** *p* < 0.01, *** *p* < 0.001

Recent studies have reported that the expression of PD-L1 is associated with tumor immune infiltration [26, 27]. To investigate the association of PD-L1 expression with immune infiltration in FTC tumors, FTC patients from the TCGA cohort were divided into high and low expression groups according to the median value of PD-L1 mRNA expression. The ESTIMATE and CIBERSORT algorithms were applied to quantify the proportions of tumor-infiltrating immune cells in FTC samples from the TCGA cohort. The ESTIMATE results illustrated that there was a positive correlation between PD-L1 expression and the immune infiltration score, PD-L1 expression was negatively correlated with tumor purity (Fig. 2a). Tumor-infiltrating immune cell analysis by CIBERSORT demonstrated that the proportions of regulatory T cells and M1 macrophages were increased in the PD-L1 high expression group, whereas the proportions of memory B cells, resting mast cells and monocyte cells were increased in the PD-L1 low expression group (Fig. 2b). Fig. S1 shows the correlation between PD-L1 expression and the abundance of immune cells. We further analysed the correlations between PD-L1 expression and the expression of inhibitory checkpoint molecules (PD-1, PD-L2, CTLA-4, TIM-3, LAG-3 and VISTA, etc.). We found that the expression levels of inhibitory checkpoint molecules, including CTLA-4, PD-L2 (PDCD1LG2), TIM3 (HAVCR2) and TIGIT, were upregulated in the PD-L1 low expression group (Fig. 2c). Subsequently, the TCIA database was used to evaluate the relationship between PD-L1 expression and the response to immune checkpoint inhibitors (CTLA-4 and PD-1 blockers) in FTC patients. The IPS in the PD-L1 high expression group was higher than that in the PD-L1 low expression group, which predicted that patients with higher PD-L1 expression had a better response to immune checkpoint inhibitors (Fig. 2d). These results suggested that PD-L1 expression was associated with tumor immune infiltration and may play a critical role in the progression of FTC.

### Functional pathway analysis of PD-L1 expression in FTC

**Fig. 3:**
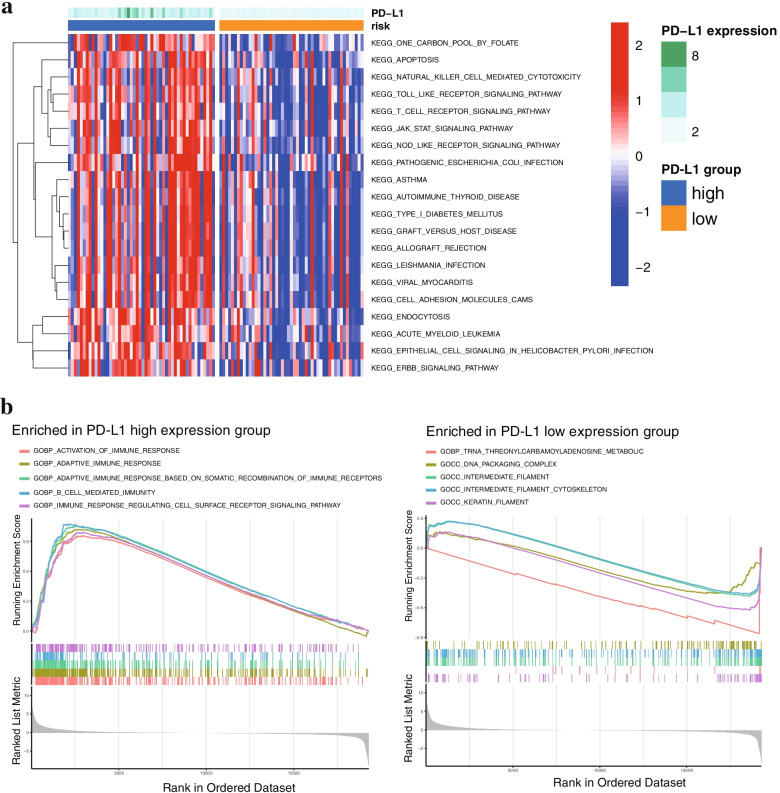
Biofunctional pathway analysis of PD-L1 expression in FTC. **a** GVSA analysis was used to identify the biological differences between the PD-L1 high and low expression groups. **b**. GSEA of GO functional pathways between the PD-L1 high and low expression groups

Further biofunctional and pathway analyses were applied to explore the difference between high and low PD-L1 expression in the TCGA cohort. GVSA analysis was used to investigate the biological differences between these two groups. Our results revealed that several pathways related to the immune response, including the Toll-like receptor signalling pathway, apoptosis, the T-cell receptor signalling pathway and the JAK/STAT signalling pathway, were enriched in the PD-L1 high expression group (Fig. 3a). Similarly, GSEA-based GO analysis demonstrated that activation of the immune response, adaptive immune response and immune response-regulating cell surface receptor signalling pathway were enriched in the PD-L1 high expression group (Fig. 3b). These results suggested that PD-L1 expression was related to the immune response pathway, which may partly explain why patients with high PD-L1 expression had a better response to immune checkpoint inhibitors.

### Silencing PD-L1 inhibited the proliferation, migration and invasion of FTC cells

**Fig. 4:**
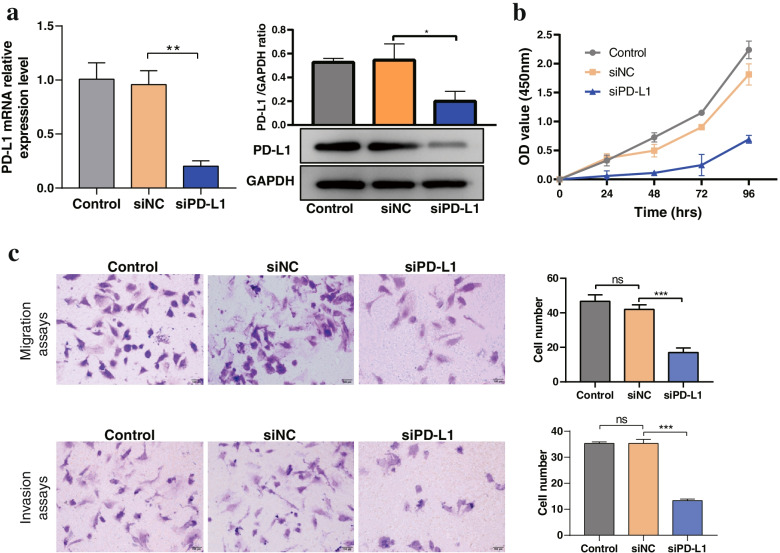
Effects of PD-L1 on the proliferation, migration and invasion of FTC cells.** a** The mRNA and protein levels of PD-L1 were detected using RT–qPCR and Western blotting, respectively. **b** A CCK-8 assay was used to detect the proliferation ability of FTC-133 cells after transfection with PD-L1 siRNA. **c** Transwell migration and invasion assays were conducted in FTC-133 cells cotransfected with siNC and the optimal siPD-L1. Data are presented as the mean ± standard error based on at least three independent experiments. * *p* < 0.05, ** *p* < 0.01, *** *p* < 0.001, ns: not significant

To investigate the potential protumorigenic role of PD-L1 in FTC, we used PD-L1 siRNA to downregulate the expression of PD-L1 in FTC-133 cells. As shown in Fig. 4a, the expression levels of PD-L1 mRNA and protein were largely downregulated after transfection with PD-L1 siRNA. Next, we used CCK-8 assays to evaluate the influence of PD-L1 on FTC-133 cell proliferation. We found that the proliferation ability of FTC-133 cells was significantly inhibited by PD-L1 siRNA transfection compared with control-siRNA transfection (Fig. 4b). Consistently, the Transwell migration and invasion assays showed that PD-L1 downregulation in FTC cells could significantly inhibit cell migration and invasion capability (Fig. 4c). Collectively, these results indicated that PD-L1 was involved in the proliferation and aggressiveness of FTC.

### MiR-199a-5p expression was downregulated in FTC tissues and cell lines

**Fig. 5:**
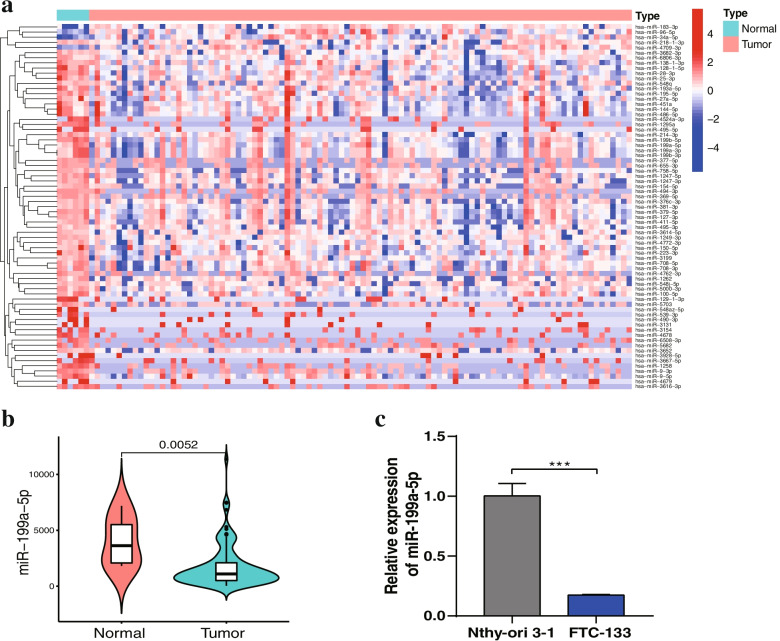
Downregulated expression of miR-199a-5p in FTC tissues and cell lines.** a** Differential expression of the miRNA heatmap in the TCGA cohort. **b** The expression of miR-199a-5p was significantly decreased in FTC tissues compared with normal tissues. **c** The expression of miR-199a-5p was downregulated in FTC-133 cells compared with Nthy-ori 3–1 cells. Data are presented as the mean ± standard error based on at least three independent experiments. * *p* < 0.05, ** *p* < 0.01, *** *p* < 0.001

An increasing number of studies have reported that miRNAs are involved in cancer progression and carcinogenesis of thyroid carcinoma [28]. Here, the TCGA cohort was used to investigate the differential expression of miRNAs between adjacent normal tissue and tumor tissues of FTC. We identified a total of 71 differentially expressed miRNAs, with 4 upregulated and 67 downregulated in FTC tissues (Fig. 5a). Among them, miR-199a-5p had the highest fold changes and greatest consistency among samples between normal and tumor tissues (Fig. 5b). Subsequently, RT–qPCR analysis was performed to detect the expression of miR-199a-5p in FTC-133 and Nthy-ori 3–1 cells. Our results showed that the miR-199a-5p expression level was downregulated in FTC-133 cells compared to Nthy-ori 3–1 cells (Fig. 5c).

### MiR-199a-5p inhibited the proliferation, migration and invasion of FTC cells

**Fig. 6:**
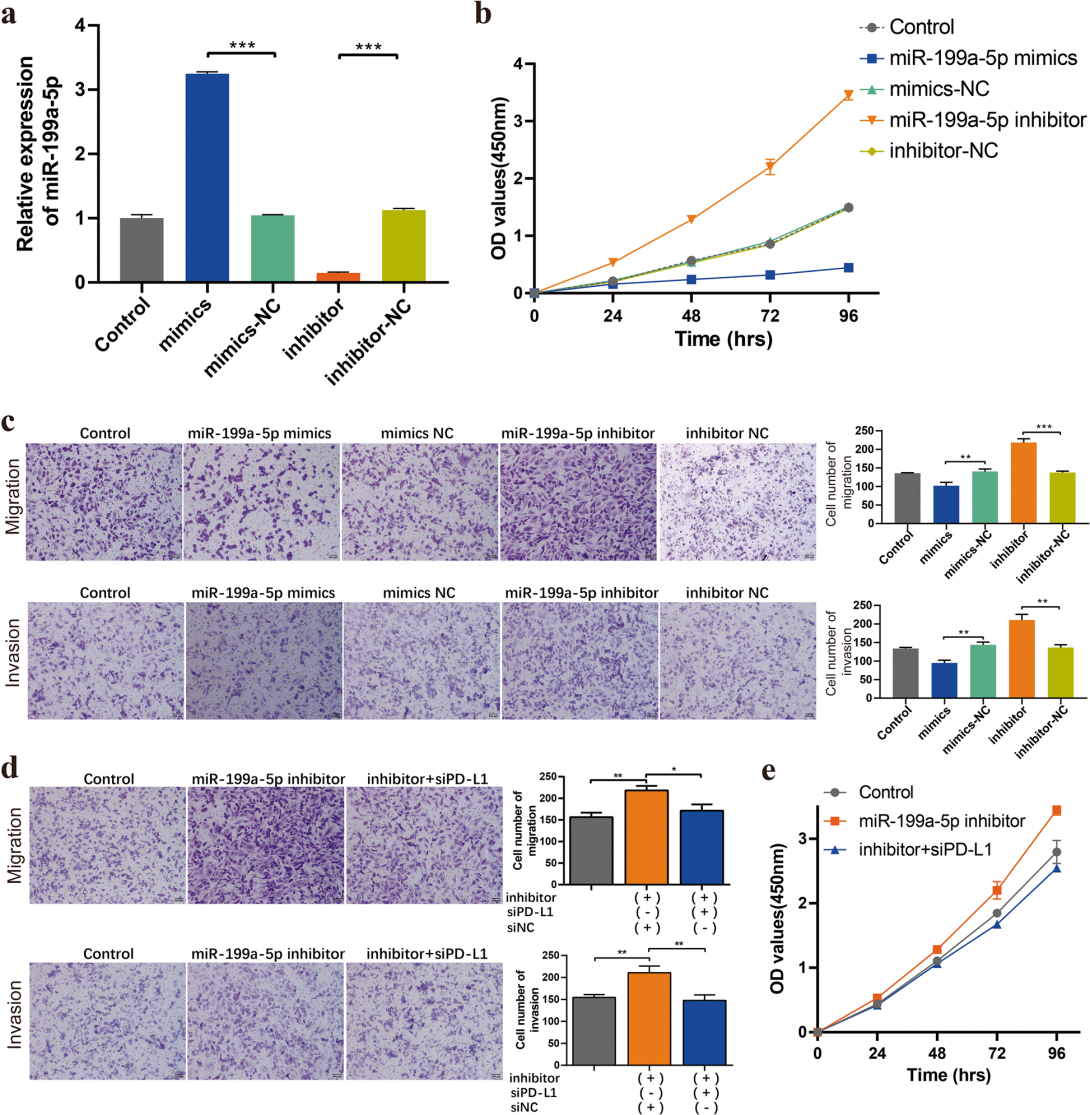
Effects of miR-199a-5p on the proliferation, migration and invasion of FTC cells.** a** The expression of miR-199a-5p in FTC cells transfected with miR-199a-5p mimics/inhibitor or negative controls. **b** CCK-8 assay was used to detect the cell proliferation ability of FTC-133 cells after transfection with miR-199a-5p mimics or inhibitor. **c** The Transwell migration/invasion assay number was detected at 72 h after FTC cells were transfected with miR-199a-5p mimics/inhibitor. **d** Knockdown of PD-L1 expression reversed the promotive effect of the miR-199a-5p inhibitor on cell migration/invasion ability and **e** cell proliferation in FTC cells. Data are presented as the mean ± standard error based on at least three independent experiments. * *p* < 0.05, ** *p* < 0.01, *** *p* < 0.001

To evaluate the biological functions of miR-199a-5p in FTC, miR-199a-5p mimics, inhibitors or corresponding negative controls were transfected into FTC-133 cells to explore its roles in the development of FTC. As shown in Fig. 6a, miR-199a-5p expression was upregulated in the miR-199a-5p mimic group and downregulated in the miR-199a-5p inhibitor group. The results of the CCK8 assay indicated that cell proliferation was inhibited by the upregulation of miR-199a-5p in FTC cells. In contrast, downregulation of miR-199a-5p promoted the proliferation of FTC cells (Fig. 6b). The Transwell migration and invasion assays indicated that overexpression of miR-199a-5p suppressed the ability of cell migration and invasion, while downregulation of miR-199a-5p enhanced the migration and invasion of FTC cells (Fig. 6c). Furthermore, rescue experiments showed that knockdown of PD-L1 partly reversed the promotive effect of the miR-199a-5p inhibitor on cell migration/invasion ability (Fig. 6d) and cell proliferation (Fig. 6e) in FTC cells. Taken together, these results showed that miR-199a-5p can inhibit the proliferation, migration and invasion of FTC cells.

### The PD-L1 gene was a direct target of miR-199a-5p

**Fig. 7:**
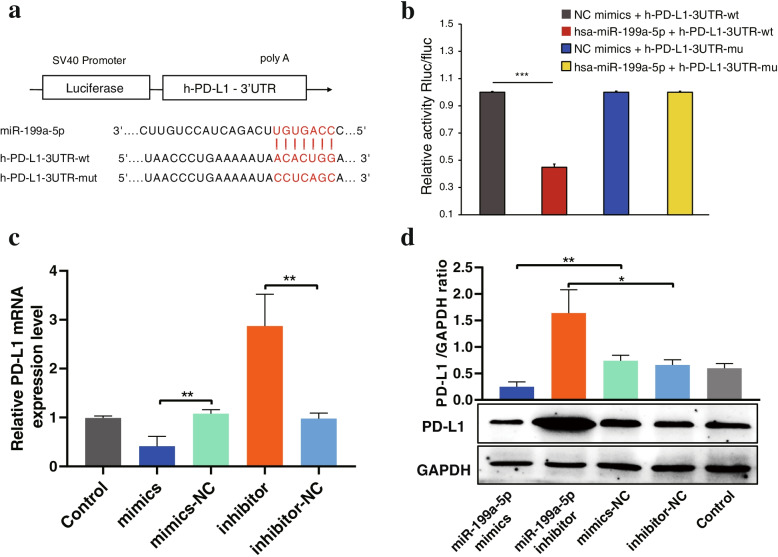
PD-L1 was a direct target gene of miR-199a-5p. **a** Schematic diagram of the binding sites of miR-199a-5p in the PD-L1 3′UTR, which was indicated with red characters. **b** Luciferase reporter assay showed that cotransfection with PD-L1 3′UTR-WT and miR-199a-5p mimics significantly reduced the luciferase activity of the WT reporter vector but not the mutant reporter vector. **c** The expression level of PD-L1 was measured by RT–qPCR and **d** western blotting after transfection with miR-199a-5p mimics or miR-199a-5p inhibitor in FTC cells. Data are presented as the mean ± standard error based on at least three independent experiments. * *p* < 0.05, ** *p* < 0.01, *** *p* < 0.001

A complementary sequence of miR-199a-5p was found in the 3′-UTR of PD-L1 according to bioinformatics analysis of the online database (Fig. 7a). Next, luciferase reporter assay was performed to verify the predicted target gene of miR-199a-5p. As we expected, the luciferase reporter assay demonstrated that miR-199a-5p mimics markedly inhibited the luciferase activity of the PDL1-3UTR-WT vector but failed to affect the luciferase activity of the PDL1-3UTR-MU vector (Fig. 7b). In addition, we found that the mRNA and protein expression levels of PD-L1 were decreased in the miR-199a-5p mimic group and markedly increased in the miR-199a-5p inhibitor group in contrast to the control group in FTC cells (Fig. 7c-d). Furthermore, we analysed the correlation between miR-199a-5p and PD-L1 expression in the TCGA cohort. However, no correlation was observed between miR-199a-5p and PD-L1 (Fig. S2a). Overall, these results indicated that PD-L1 was a direct target gene of miR-199a-5p.

### PD-L1 and Claudin-1 expression levels were positively correlated in FTC

**Fig. 8:**
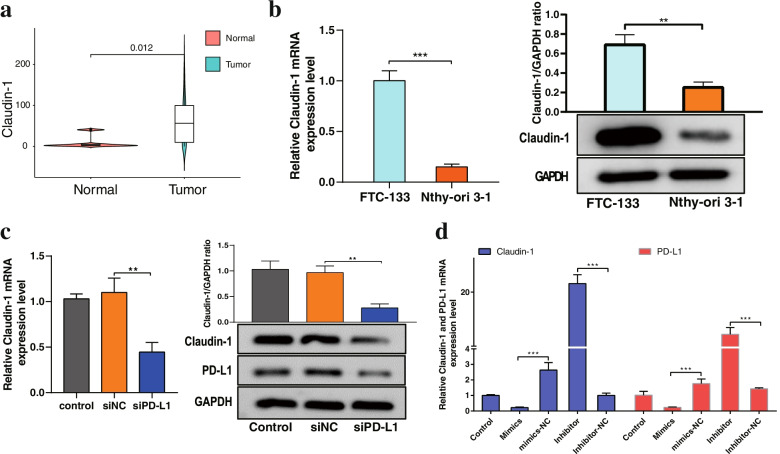
PD-L1 and Claudin-1 expression was positively correlated in FTC.** a** The expression of Claudin-1 was increased in FTC tissues compared with normal tissues. **b** The mRNA and protein expression levels of Claudin-1 were examined by RT–qPCR and Western blotting, respectively. **c** The expression level of Claudin-1 was measured by RT–qPCR and Western blotting after transfection with PD-L1 siRNA in FTC cells. **d** The mRNA expression levels of Claudin-1 and PD-L1 were measured by RT–qPCR after transfection with miR-199a-5p mimics or miR-199a-5p inhibitor in FTC cells. Data are presented as the mean ± standard error based on at least three independent experiments. * *p* < 0.05, ** *p* < 0.01, *** *p* < 0.001

The overexpression of Claudin-1 has been reported to correlate with cell proliferation and aggressiveness in thyroid carcinoma. We next analysed Claudin-1 expression in FTC tumors from the TCGA database. We found that the expression level of Claudin-1 was upregulated in tumor tissue compared to adjacent normal tissues (Fig. 8a). This result was also consistent with the in vitro study, which showed that the expression levels of Claudin-1 mRNA and protein were significantly higher in FTC-133 cells than in Nthy-ori 3–1 cells (Fig. 8b). As mentioned above, we further investigated the relationships between PD-L1 and Claudin-1, which were highly expressed and associated with tumor aggressiveness in FTC. Interestingly, our results revealed that PD-L1 expression was positively correlated with Claudin-1 expression in FTC cells. Knockdown of PD-L1 significantly downregulated the expression of Claudin-1 (Fig. 8c). Additionally, miR-199a-5p upregulation suppressed PD-L1 and Claudin-1 expression, while the downregulation of miR-199a-5p promoted PD-L1 and Claudin-1 expression in FTC cells (Fig. 8d). However, correlation analysis revealed that there was no significant correlation between the expression of miR-199a-5p, PD-L1 or Claudin-1 (Fig. S2b). Nevertheless, these findings suggested that miR-199a-5p affected the progression of FTC cells through negative regulation of PD-L1 and Claudin-1.

## Discussion

Recent studies have reported that PD-L1 is highly expressed in a subset of patients with advanced thyroid cancer, including FTC, and correlates with a higher risk of recurrence and shortened disease-free survival [12, 29]. In this study, we investigated the biological roles of PD-L1 in tumor progression and further identified miRNAs that were responsible for regulating the activity of PD-L1 in FTC. Our data demonstrated that overexpression of PD-L1 promoted cell growth, migration and invasion in FTC, and miR-199a-5p inhibited the proliferation, migration and invasion of FTC cells by regulating the expression of PD-L1 and Claudin-1.

PD-L1 is a costimulatory molecule expressed on the surfaces of tumor cells and immune cells that plays a pivotal role in the immune system [19]. However, PD-L1 can also influence tumor progression by regulating various tumor-intrinsic events of tumor cells independent of the immune system [30]. Our study confirmed that PD-L1 can enhance proliferation, migration and invasion in FTC cells, which displayed its tumor-intrinsic functions independent of its immunopathogenic effects. Therefore, the high expression of PD-L1 may be correlated with an increased degree of malignancy and poor prognosis of the tumor. However, there was no significant difference in the expression level of PD-L1 mRNA between normal and tumor samples in the TCGA cohort, possibly because many processes, including transcription and translation, occur between the production of PD-L1 mRNA and protein, and gene expression sometimes cannot be interpreted in terms of protein levels.

The immune system plays a crucial role in the recognition and elimination of tumor cells. The interaction of PD-1/PD-L1 plays important roles in the maintenance of immunological tolerance and in modulating the immune response [31]. Emerging evidence has revealed that the expression of PD-L1 on tumor cells leads to immunosuppression and consequently enhances aggressiveness [19, 32]. Therefore, PD-L1 expression has been considered a potential biomarker for the response to anti-PD-1/PD-L1 agents in various tumors [11, 33]. Immune infiltration profiling of the tumor microenvironment is often applied to determine a patient’s potential for response to immunotherapy [34, 35]. Thyroid carcinomas are generally considered to be one of the most “hot” tumor types [36]. There were different cut-off values of PD-L1 protein expression for predicting immunotherapy responses in different types of tumors, which were assessed by immunochemistry. However, the appropriate cut-off value of PD-L1 mRNA expression is still not explicit. In our study, FTC patients were divided into high and low PD-L1 expression groups according to the median value of PD-L1 mRNA expression. We found that PD-L1 expression was positively correlated with immune infiltration, especially regulatory T cells and M1 macrophages. Regulatory T cells play a requisite role in the maintenance of immunological homeostasis, and M1 macrophages represent a highly proinflammatory subset of macrophages [37]. These data may indicate that FTC patients with high PD-L1 expression respond well to immunotherapy.

Over 30% of human genes are regulated by miRNAs, and loss of expression or mutation of miRNAs may lead to serious diseases, including cancer [38]. MiRNAs have been proven to have multiple roles in regulating tumor proliferation, development and metastasis [39]. Many studies have revealed that miRNAs such as miR-199a-5p, miR-146b and miR-183-5p are deregulated in thyroid cancer and can act as potential biomarkers in distinguishing thyroid malignancy [40–42]. In this study, we found that miR-199a-5p expression was downregulated in FTC tissue and cell lines. The expression of miR-199a-5p was found to be diversely deregulated in different types of tumors. Qu et al. found that miR-199a-5p can promote cell proliferation and metastasis of cervical cancer [43]. Chen et al. reported that miR-199a-5p can significantly inhibit cell migration and invasion in breast cancer [44]. Several reports have revealed that miR-199a-5p plays crucial roles in the development and progression of thyroid carcinoma [45, 46]. Our data are consistent with previous reports that miR-199a-5p suppresses proliferation, migration and invasion in FTC cells [46]. At the same time, we also proved that PD-L1 was a direct target of miR-199a-5p. These results suggested that miR-199a-5p may inhibit the progression of FTC by targeting PD-L1. Indeed, the rescue experiments confirmed that knockdown of PD-L1 could reverse the miR-199a-5p inhibitor mediated promotion of FTC cell proliferation, migration and invasion. These results indicated that miR-199a-5p may be a potential tumor suppressor gene to inhibit the biological activity of FTC cells. The mechanism by which miR-199a-5p targets PD-L1 to regulate the biological characteristics of FTC-133 cells remains unclear. Sun et al. reported that downregulation of miR-199a-5p upregulated the expression of CTGF and promoted the viability of the cells by increasing the fractions of cells in the G2/M and S phases [46]. Tumor cells can acquire the ability to invade surrounding tissues because of the disruption to cell–cell adhesion. Denise et al. reported that overexpression of Claudin-1 was associated with increased migration and invasion of follicular thyroid carcinoma cells [47]. Our study showed that PD-L1 and Claudin-1 were significantly upregulated with miR-199a-5p knockdown, while miR-199a-5p overexpression downregulated PD-L1 and Claudin-1. These results indicated that miR-199a-5p can regulate the expression of Claudin-1 via PD-L1 in FTC. Xiong et al. showed that STAT3-Y705 can be phosphorylated in response to hypoxia and then p-Y705-Stat3 may bind to PD-L1 [48]. Meanwhile, Chen et al. found that the expression of Claudin-1 may be reduced by the phosphorylation (activation) of STAT3 and the expression of Claudin-1 could be promoted by inhibiting STAT3 phosphorylation in hepatocellular carcinoma [49]. A mechanism may be involved that phosphorylated-STAT3 binds to the PD-L1 promoter and activates its transcription. Taken together, the results suggest that phosphorylated-STAT3 may be combined to the PD-L1, and then losing its inhibitory effect on Claudin-1, resulting the over expression of Claudin-1.

However, there were several limitations in this study. First, our study did not investigate the potential signalling pathways of miR-199a-5p and PD-L1 in the regulatory mechanisms of FTC. Further experimental research is needed to explore the underlying molecular mechanism. Second, animal models should be used to confirm these results and clarify the biological functions.

## Conclusions

Taken together, our study demonstrated that the proportion of PD-L1 positive samples was higher in FTC tissues than in normal tissues. Knockdown of PD-L1 expression significantly suppressed the proliferation, migration and invasion of FTC cells. miR-199a-5p affects the progression and metastasis of FTC cells through the negative regulation of PD-L1 and Claudin-1.

## Supplementary Information

**Additional file 1:** **Table S1.** The sequences of siRNA and miRNA. **Table S2.** Sequences of the primer used for RT-qPCR. **Fig. S1.** Correlation of PD-L1 expression with tumor infiltrating immune cells in TCGA. **Fig. S2.** a Correlation analysis between PD-L1 with miR-199a-5p expression in TCGA cohort. b Correlation analysis between Claudin-1 with miR-199a-5p, PD-L1 expression in TCGA cohort.

**Additional file 2.**

## Data Availability

The datasets analyzed for this study can be found in the TICA (https://www.tcia.at/home) and TCGA (https://portal.gdc.cancer.gov/).

## Abbreviations

TC: Thyroid carcinoma
DTC: Differentiated thyroid carcinoma
ATC: Undifferentiated thyroid cancer
MTC: Medullary thyroid carcinoma
PTC: Papillary thyroid carcinoma
FTC: Follicular thyroid carcinoma
IHC: Immunohistochemical
TPS: Tumor proportion score
FBS: Fetal bovine serum
FC: Foldchange
PVDF: Polyvinylidene difluoride membranes
CCK-8: The Cell Counting Kit-8
IPS: Immunophenoscores
