# Supplementary material for: The miR-199a-5p/PD-L1 axis regulates cell proliferation, migration and invasion in follicular thyroid carcinoma
Source: BMC Cancer. 2022 Jul 11;22:756. doi: 10.1186/s12885-022-09838-0 (PMC9275143; doi:10.1186/s12885-022-09838-0)
Supplement: Supplementary file 1 — Additional file 1: Table S1. The sequences of siRNA and miRNA. Table S2. Sequences of the primer used for RT-qPCR. Fig. S1. Correlation of PD-L1 expression with tumor infiltrating immune cells in TCGA. Fig. S2. a Correlation analysis between PD-L1 with miR-199a-5p expression in TCGA cohort. b Correlation analysis between Claudin-1 with miR-199a-5p, PD-L1 expression in TCGA cohort. [file 12885_2022_9838_MOESM1_ESM.docx]

**Table S1 The sequences of** **siRNA and miRNA**

| Fragment | Target sequences |
| --- | --- |
| siPD-L1-1 | 5＇-CGAAUUACUGUGAAAGUCAAUTT-3＇ |
| siPD-L1-2 | 5＇-GGCAUUUGCUGAACGCAUUUATT-3＇ |
| siPD-L1-3 | 5＇-UGGAGGAUAAGAACAUUAUUCTT-3＇ |
| NC siRNA | 5＇-UUCUCCGAACGUGUCACGUTT-3＇ |
| miR-199a-5p mimics | 5＇-CCCAGUGUUAGACUACCUGUUC-3＇ |
| miR-199a-5p inhibitor | 5＇-GAACAGGUAGUCUGAACACUGGG-3＇ |
| mimics NC | 5＇-UUGUACUACACAAAAGUACUG-3＇ |
| inhibitor NC | 5＇-CAGUACUUUUGUGUAGUACAA-3＇ |

**Table S2** **Sequences of the primer used for RT-qPCR**

| genes | Target sequences |
| --- | --- |
| PD-L1 | Forward 5＇-GCTGCACTAATTGTCTATTGGG-3＇ |
|  | Reverse 3＇-CACAGTAATTCGCTTGTAGTCG-5＇ |
| Claudin-1 | Forward 5＇-AGATACAGTGCAAAGTCTTCGA-3＇ |
|  | Reverse 3＇-CAGGATGCCAATTACCATCAAG-5＇ |
| Occludin | Forward 5＇-TGCTTCATCGCTTCCTTAGTAA-3＇ |
|  | Reverse 3＇-GGGTTCACTCCCATTATGTACA-5＇ |
| GAPDH | Forward 5＇-ACCACAGTCCATGCCATCAC-3＇ |
|  | Reverse 3＇-TCCACCACCCTGTTGCTGTA-5＇ |


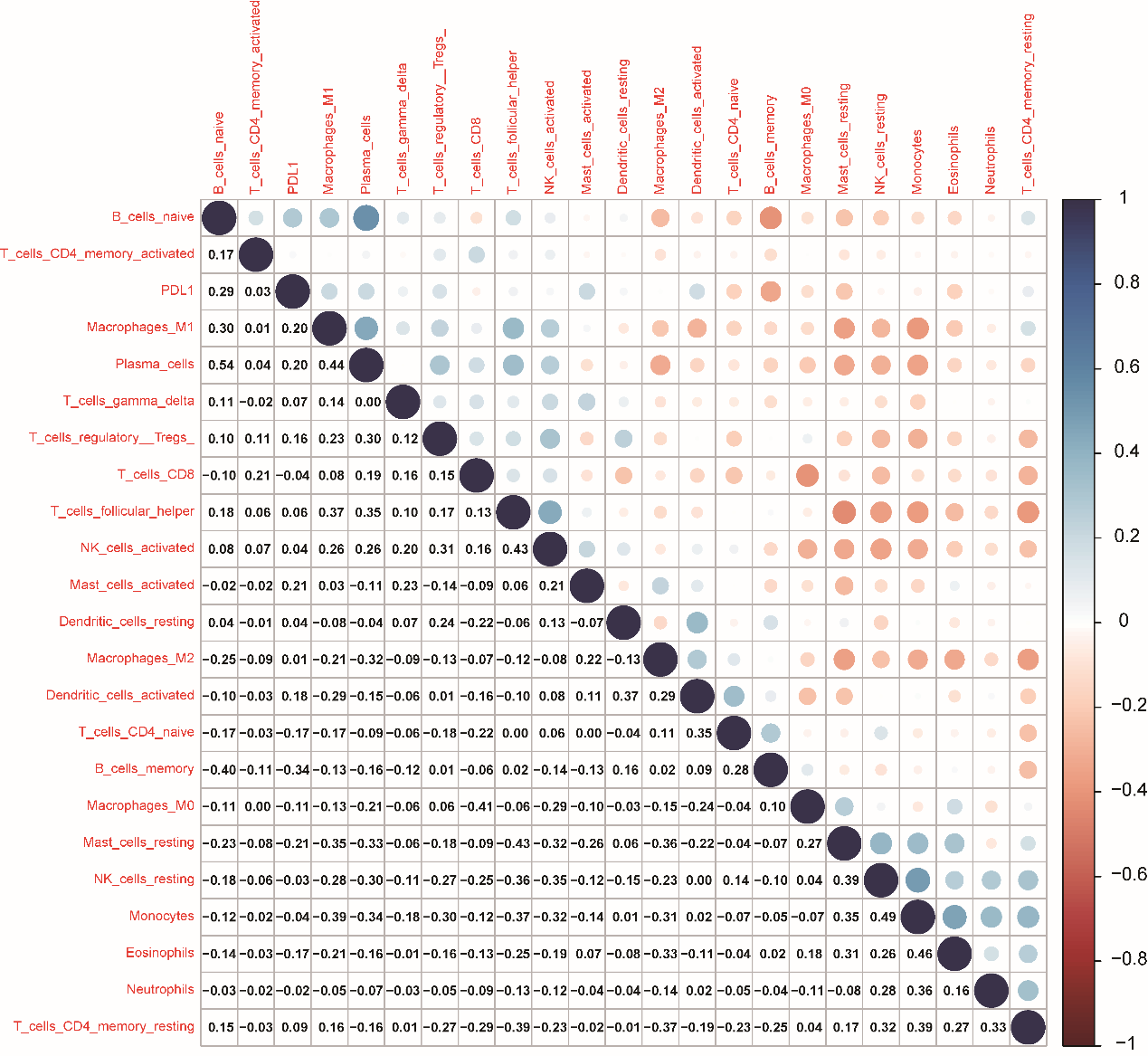


**Fig. S1** Correlation of PD-L1 expression with tumor infiltrating immune cells in TCGA.


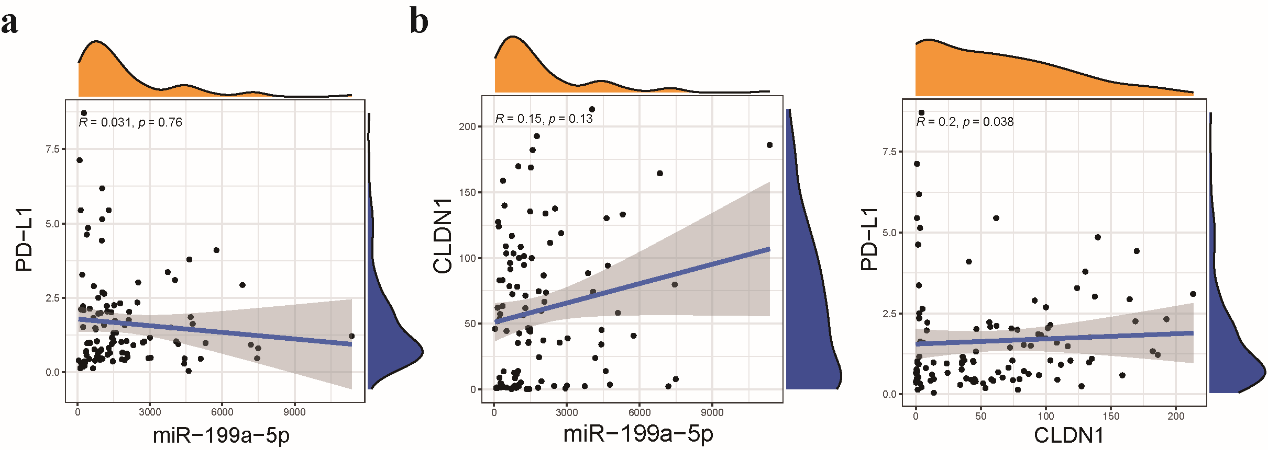


**Fig. S2 a** Correlation analysis between PD-L1 with miR-199a-5p expression in TCGA cohort. **b** Correlation analysis between Claudin-1 with miR-199a-5p, PD-L1 expression in TCGA cohort.
