# Supplementary figures and images for: The miR-199a-5p/PD-L1 axis regulates cell proliferation, migration and invasion in follicular thyroid carcinoma
Source: BMC Cancer. 2022 Jul 11;22:756. doi: 10.1186/s12885-022-09838-0 (PMC9275143; doi:10.1186/s12885-022-09838-0)

Fig 1C


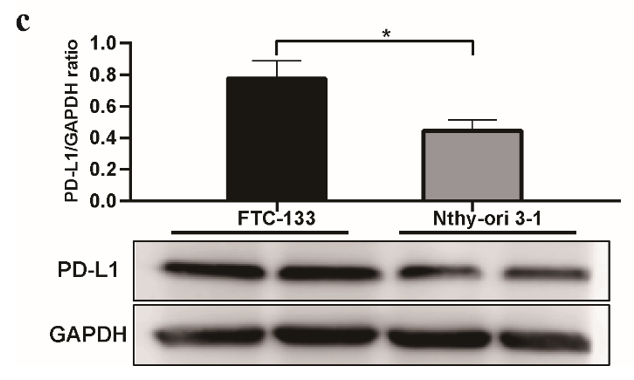







Fig 4A


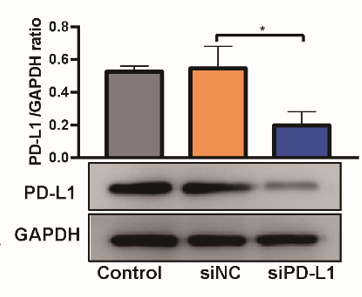

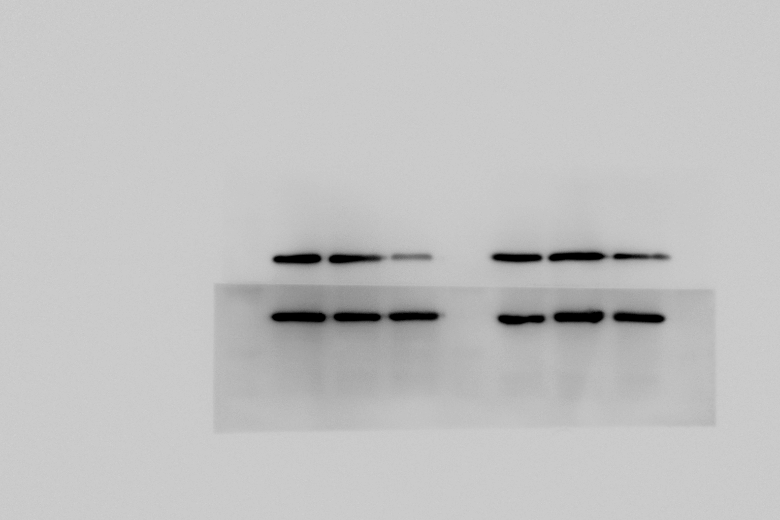


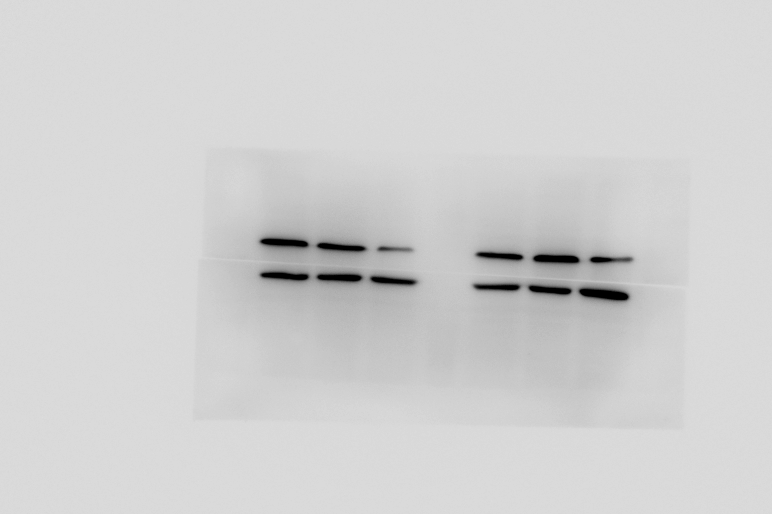


Fig 7D


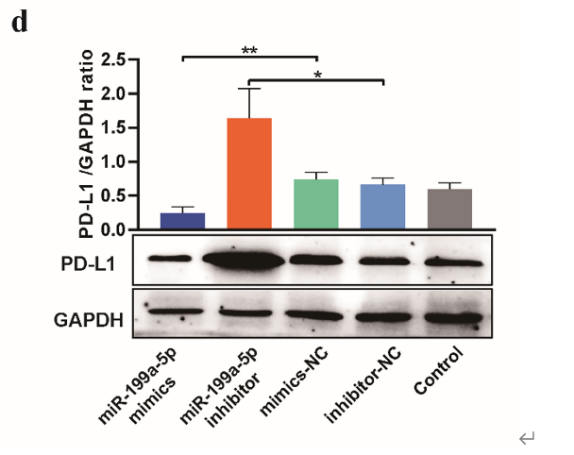

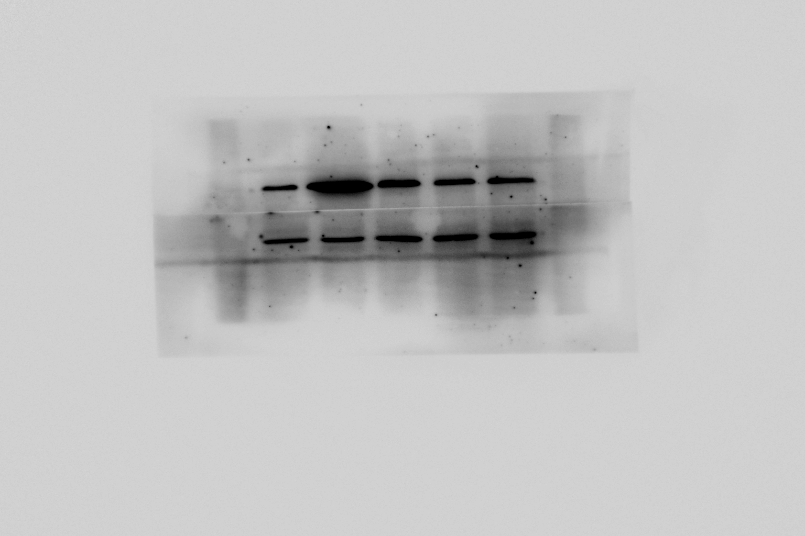


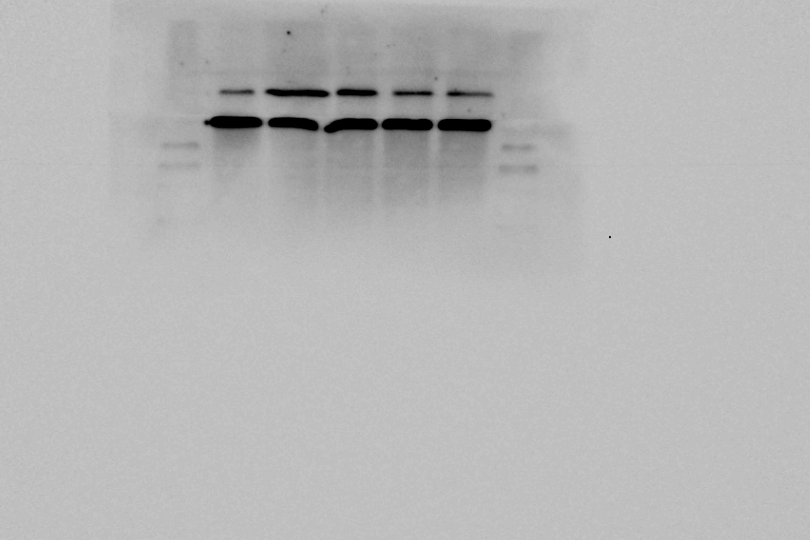


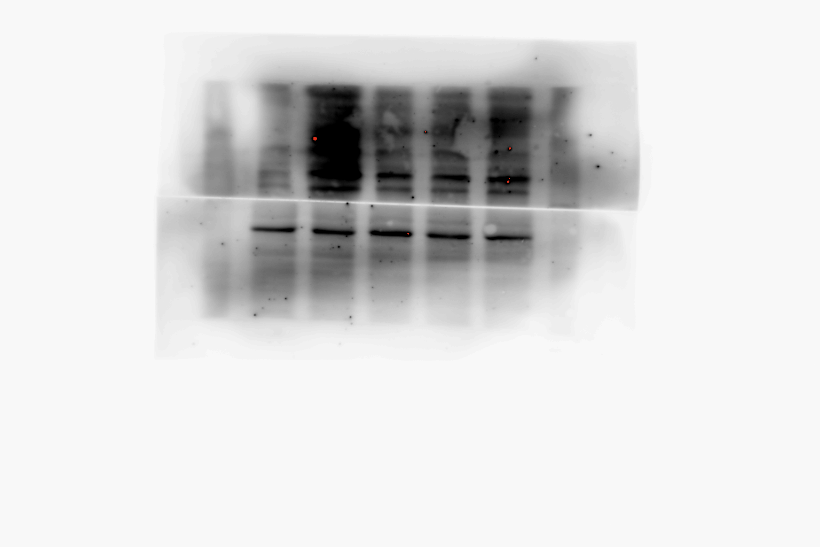


Fig 8B


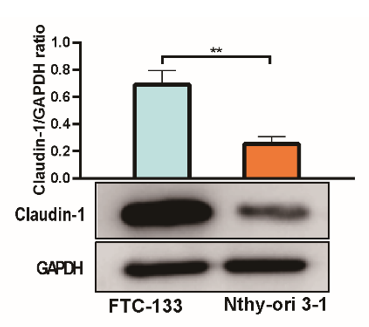

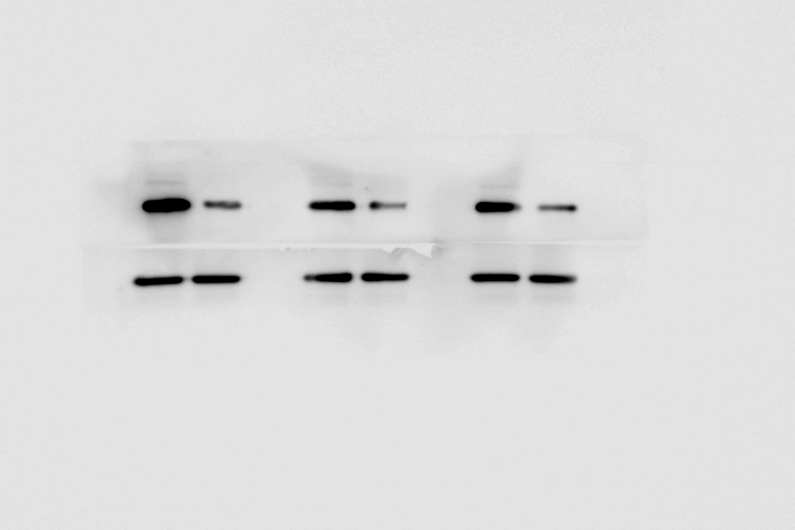





Fig 8C

**
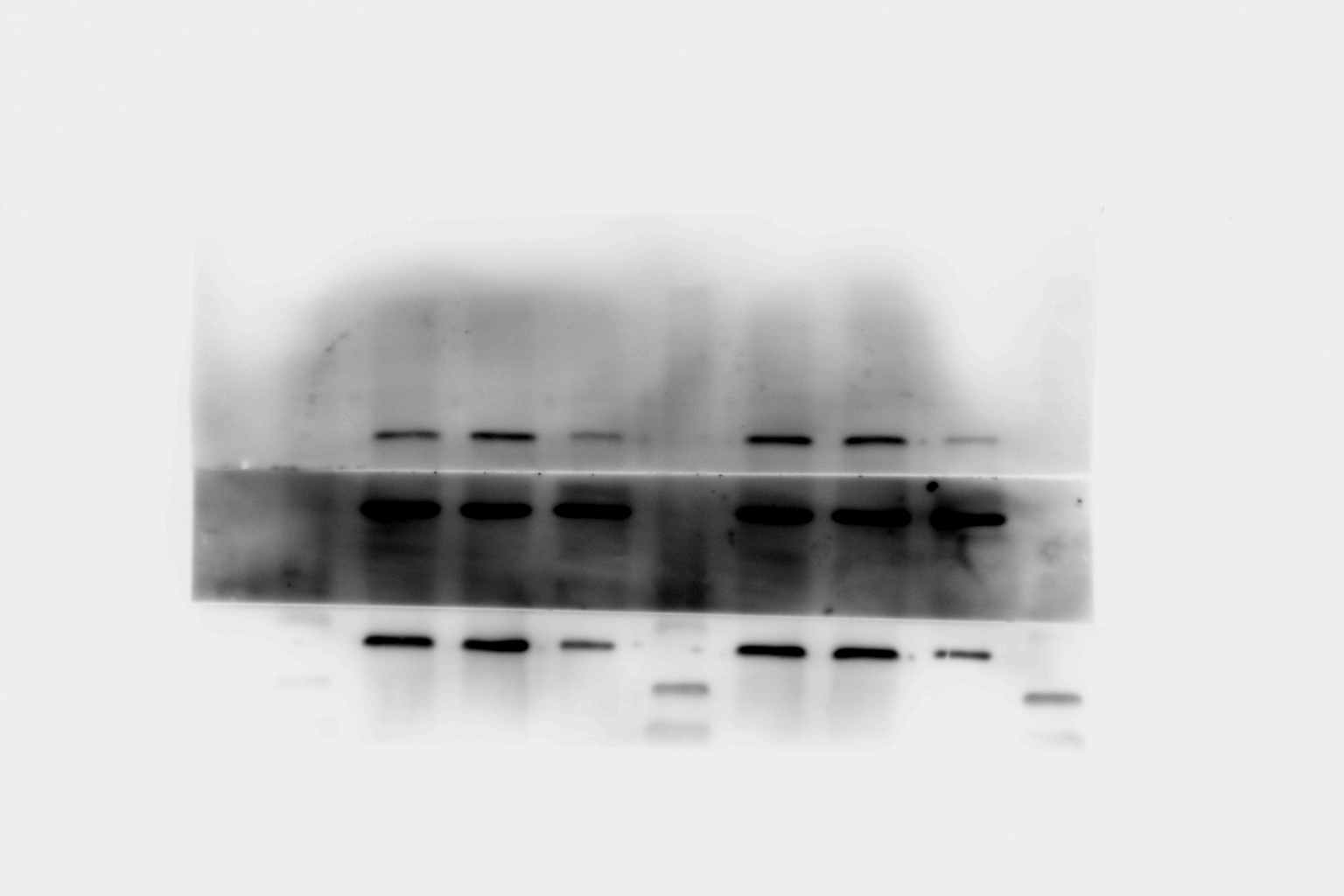
**

Claudin-1

GAPDH

PD-L1


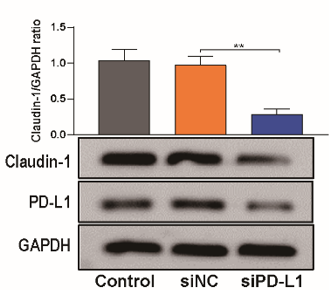

Supplement: Supplementary file 2 — Additional file 2. [file 12885_2022_9838_MOESM2_ESM.docx]
